# The Synthetic Retinoid Acitretin Increases IL-6 in the Central Nervous System of Alzheimer Disease Model Mice and Human Patients

**DOI:** 10.3389/fnagi.2019.00182

**Published:** 2019-07-23

**Authors:** Malena dos Santos Guilherme, Nicolai M. Stoye, Stefan Rose-John, Christoph Garbers, Andreas Fellgiebel, Kristina Endres

**Affiliations:** ^1^Department of Psychiatry and Psychotherapy, University Medical Center, Johannes Gutenberg University Mainz, Mainz, Germany; ^2^Institute of Biochemistry, Christian-Albrechts-Universität zu Kiel (CAU Kiel), Kiel, Germany; ^3^Department of Pathology, Medical Faculty, Otto-von-Guericke-University Magdeburg, Magdeburg, Germany

**Keywords:** alpha-secretase, ADAM10, gp130, IL-6, IL-6R, inflammation, retinoic acid, vitamin A

## Abstract

These days, the important role of retinoids in adult brain functionality and homeostasis is well accepted and has been proven by genomic as well as non-genomic mechanisms. In the healthy brain, numerous biological processes, e.g., cell proliferation, neurogenesis, dendritic spine formation as well as modulation of the immune system, have been attributed to retinoid signaling. This, together with the finding that retinoid metabolism is impaired in Alzheimer’s disease (AD), led to preclinical and early clinical testing of natural and synthetic retinoids as innovative pharmaceuticals with multifactorial properties. Acitretin, an aromatic retinoid, was found to exert an anti-amyloidogenic effect in mouse models for AD as well as in human patients by stimulating the alpha-secretase ADAM10. The lipophilic drug was already demonstrated to easily pass the blood brain barrier after i.p. administration and evoked increased nest building capability in the 5xFAD mouse model. Additionally, we analyzed the immune-modulatory capacity of acitretin *via* a multiplex array in the 5xFAD mouse model and evaluated some of our findings in human CSF derived from a pilot study using acitretin. Although several serum analytes did not display changes, Interleukin-6 (IL-6) was found to be significantly increased in both—mouse and human neural material. This demonstrates that acitretin exerts an immune stimulatory effect—besides the alpha-secretase induction—which could impact the alleviation of learning and memory disabilities observed in the mouse model.

## Introduction

In a meta-analysis, significantly lower vitamin A plasma levels were found in Alzheimer’s disease (AD) patients (Lopes da Silva et al., 2014) and genetic linkages in regard to retinoic acid receptors as well as altered enzymatic function e.g., for retinaldehyde dehydrogenase (Connor and Sidell, 1997; Goodman and Pardee, 2003). Pre-clinical data support that retinoids might contribute to development and progression of AD: for example, vitamin A deficiency evoked in adult rodents cognitive impairment (Jiang et al., 2012; Hou et al., 2015) and a shift towards the amyloidogenic processing of the amyloid precursor protein (APP; Reinhardt et al., 2016). Additionally, marginal vitamin A deficiency (MVAD) that has also been correlated with cognitive decline in the elderly, led to enhanced Aβ synthesis, plaque formation, and cognitive deficits in APP/PS1 mice (Zeng et al., 2017a). Moreover, rats fed on a MVAD diet with combinatory injection of Aβ showed cognitive impairment (Zeng et al., 2017b). This indicates that retinoic acid deficiency may lead to enhanced risk of developing AD and has given rise to the idea of using retinoids as therapeutics (Fahrenholz et al., 2010; Chakrabarti et al., 2016).

Severe unwanted side-effects from systemic retinoid-intake mainly comprise teratogenicity (Hunt, 1996), which should not be highly relevant in patients aged over 60 (beyond childbearing age). Nevertheless, retinoids can also lead to symptoms such as bone fractures (Green et al., 2016), which would contribute to frailty in the aged patients. Moreover, retinoids might also exert psychiatric side-effects such as those reported for isotretinoin application in acne-treatment and depression or aggression (Bremner et al., 2012), thereby further substantiating their importance for neuronal network stability. This makes it absolutely mandatory to monitor side-effects and identify further potential molecular targets of retinoid-treatment. We hypothesized that treatment with acitretin, a synthetic retinoid that already has proven anti-amyloidogenic effect in AD patients (Endres et al., 2014), would lead to modifications in peripheral and central immunity where retinoids have been identified as potent regulators (reviewed in Erkelens and Mebius, 2017). This can consequently also impact retinoic acid metabolism in a sort of vicious cycle: for example, pro-inflammatory activation can result in increased retinoic acid catabolism as demonstrated for primary mouse microglia (Hellmann-Regen et al., 2013). Therefore, we analyzed immunological markers in serum and brain samples from AD model mice treated with acitretin and evaluated the finding of elevated brain Interleukin-6 (IL-6) in human CSF samples.

## Materials and Methods

### Animals

5xFAD mice (Jackson Laboratory; Oakley et al., 2006) stably cross-bred with C57Bl6/J mice and wild type littermates were used at an age of 30 weeks (all female, for housing conditions and genotyping see Brandscheid et al., 2017). All experimental procedures were carried out in accordance with the European Communities Council Directive regarding care and use of animals for experimental procedures and was approved by local authorities (LUA Rhineland-Palatinate; G14-1-087).

### Treatment of Mice

**Figure 1:**
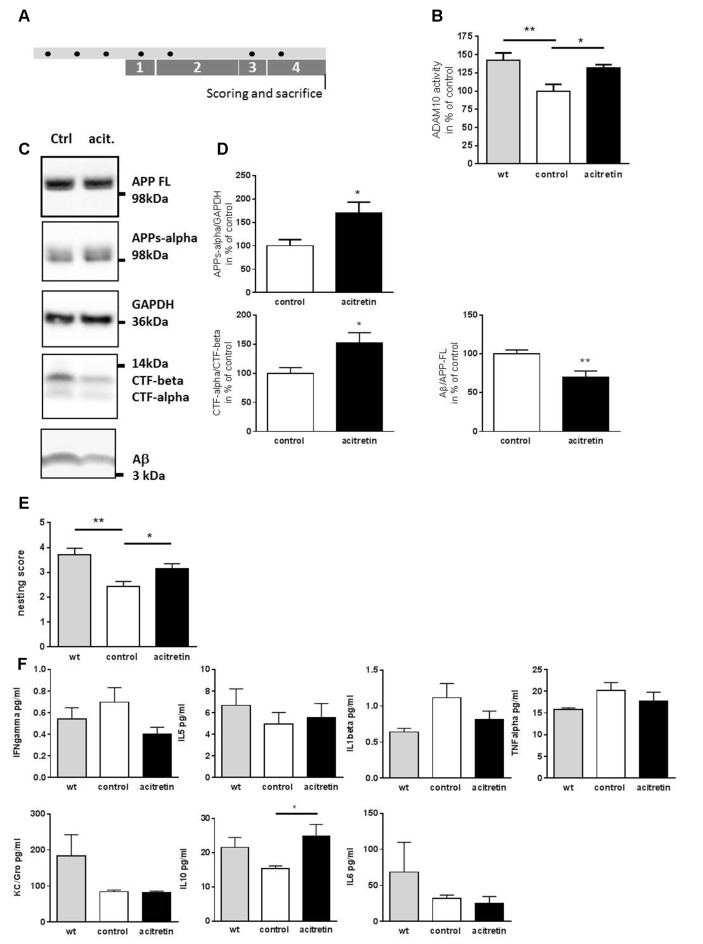
Effect of acitretin on alpha-secretase activity, nest building ability, and immunological serum markers in 5xFAD mice. Mice were treated as depicted in the scheme (**A**, black dot: injection) with a daily dosage of acitretin or with corn oil as solvent-control. As a control, wild type littermates injected with corn-oil were included. In parallel, the nesting test was conducted (1, habituation to nesting material; 2, habituation to nesting material as the sole bedding; 3, depletion of nesting material; 4, nest-building). **(B)** ADAM10 catalytic activity was measured in brain homogenates using a FRET-dependent assay. Values obtained for control-treated animals were set to 100%, mean + SEM are presented (for each group: *n* = 4; Student’s unpaired *t*-test; *p ≤ 0.05; **p ≤ 0.01). **(C,D)** Amyloid precursor protein (APP) processing products were measured by western blot and normalized to GAPDH (APPs-alpha) or to full length APP (Aβ). For CTFs, a ratio was calculated without further normalization. Values obtained for control-treated animals were set to 100%, mean + SEM are presented (*n* = 3–5 for control, *n* = 4–5 for acitretin; Student’s unpaired *t*-test; *p ≤ 0.05). **(E)** Nests were scored following a rating scale (for each group: *n* = 7–8; Mann Whitney test; *p ≤ 0.05; **p ≤ 0.01). **(F)** Analysis of peripheral immune markers by multiplex analysis. Serum samples from *n* = 4–6 animals were analyzed (one way ANOVA; Sidaks multiple comparison test; *p ≤ 0.05).

Mice were injected intraperitonally [daily dosage: 10 mg acitretin (LGC Promochem) in corn oil (Sigma Aldrich)/kg] for 7 days including a 2-day break (see treatment schedule, Figure 1A).

### Nesting Test

Nest building ability was assessed as modified from Reinhardt et al. (2018) and scored as follows: 0 = material unused; 1 = material used but not collected; 2 = material collected; 3 = nest with low walls; 4 = nest with walls as high as the mouse; 5 = walls higher than the mouse (full dome, newly introduced score level); 6 = closed dome.

### ADAM10 Activity Assay

A fluorescent enzyme assay (Sensolite 520 ADAM10 Activity Assay, Anaspec) was used according to the manufacturer’s recommendations. Brains were homogenized in ice-cold PBS supplemented with protease inhibitor cocktail (without EDTA, Roche). Homogenates were centrifuged at 3,000 *g* (3 min, 4°C); the resulting pellet was washed and resuspended with assay buffer. For each sample, a solvent control and a GM6001-treatment were measured at 480/520 (exc./em.) using the FluostarOmega (BMG; 1 measurement per minute). Usage of the metalloprotease inhibitor GM6001 ascertains control for unspecific signals obtained by the *in vitro* assay. Forty minutes from the linear range were used to calculate the fluorescence increase per minute. Specific RFU (relative fluorescence units) were calculated by subtracting the values from each GM6001-treatment sample from its corresponding solvent control.

### APP Processing Product Quantitation

Brain samples were prepared as described before for APPs-alpha quantitation (Reinhardt et al., 2016). For APP full length, CTFs and Aβ, homogenates from activity assay were used. Twenty micrograms of protein were subjected to SDS polyacrylamide gel electrophoresis. Proteins were blotted onto nitrocellulose membrane and blocked with 0.2% I-Block (Thermo Fisher Scientific) solution including 0.05% Tween20. As primary antibodies 6E10 (Covance, Madison, WI, USA), 6687 (APP CT, Steiner et al., 2000), and anti-GAPDH (14C10, Cell Signaling, Danvers, MA, USA) were used in combination with respective secondary antibody coupled with horseradish peroxidase (Thermo Scientific, Karlsruhe, Germany). Signals were obtained by SuperSignal West Femto chemiluminescent substrate (Thermo Scientific, Karlsruhe, Germany) and exposure in a CCD-camera imaging system (Raytest, Straubenhardt, Germany). Densitometric analysis was performed by Aida Image Analyzer v4.26 (Raytest).

### MSD Multiplex Array for Cytokine/Interleukin Serum Level Quantitation

The V-PLEX Proinflammatory Panel 1 Mouse Kit (Sulfo-tag antibodies, K15048D-1, Meso Scale Discovery) was used as recommended by the vendor with mouse serum diluted 1:2.75. IL-12p70, IL-2, and IL-4 from this multiplex assay were not detected in sufficient amounts (below LOD) to perform analysis.

### Interleukin-6 Measurement

Brain homogenates were centrifuged for 10 min at 10,000 *g*, and at 4°C. The supernatant was used for IL-6 measurement following the manufacturer’s protocol (IBL International, Hamburg, Germany). Values were normalized to protein content of the supernatant.

### Quantitation of IL-6R and gp130

Quantitation of IL-6R and gp130 was done *via* ELISA (R&D Systems) and normalized to the protein amount of the tissue lysate.

### Clinical Study

The acitretin-treatment study has been described in detail in Endres et al. (2014). In brief, men as well as women aged over 50 years with mild to moderate dementia and a diagnosis of probable AD were randomized to either placebo or acitretin group. Thirty milligrams of acitretin were taken daily. CSF was collected at two time points: before start of treatment (“baseline”) and after 30 days (“treatment”). IL-4 and -6 in CSF were analyzed by ELISA following the manufacturer’s instructions (IBL International, Hamburg, Germany). The study is registered with ClinicalTrials.gov (NCT01078168). Patients provided written informed consent before enrolment.

### Statistics

Testing of statistical significance was performed using one way ANOVA followed by appropriate post-test or by unpaired Student’s *t*-test (Graph Pad Prism6, San Diego, CA, USA). In case of the nesting test (ordinal scale), Mann Whitney test was used.

## Results

### Acitretin Activates ADAM10 in the Brain of 5xFAD Model Mice and Ameliorates Cognitive Deficits

In a previous study, a single stereotactic acitretin-injection was sufficient to balance APP processing towards the non-amyloidogenic pathway in APP/PS1 AD model mice (Tippmann et al., 2009). Here, we wanted to examine if peripheral (i.p.) injection in the 5xFAD mouse model also suffices to achieve this effect. We treated 30-week-old 5xFAD mice with acitretin over 10 days (treatment schedule shown in Figure 1A): first, ADAM10 activity within brain homogenates of 5xFAD mice was decreased by ca. 40% as compared to wild type littermates (Figure 1B). This deficiency could be partially restored by acitretin-administration (131% of control-treated 5xFAD, Figure 1B) and was accompanied by increased shedding of APPs-alpha, increased CTF-alpha/-beta ratio, and decreased amount of Aβ (Figures 1C,D). Moreover, acitretin-treatment was able to ameliorate cognitive impairment as demonstrated by nest building test (Figure 1E).

### Acitretin Has Only Minor Effect on Peripheral Immune Markers but Increases Cerebral IL-6 Amount

No significant differences in serum interferon gamma, IL-5, KC/Gro, TNF alpha, and IL-1beta could be measured in acitretin-treated vs. control-injected animals (Figure 1F). Only anti-inflammatory IL-10 increased significantly, reaching similar levels as in wild type littermates. All-trans retinoic acid has been shown to suppress IL-6, an important cytokine of the aging brain (Godbout and Johnson, 2004) *via* ERK1/2 activation (Kirchmeyer et al., 2008) and we could see non-significant reduction of IL-6 in the peripheral samples (32 vs. 25 pg/ml, Figure 1F).

**Figure 2:**
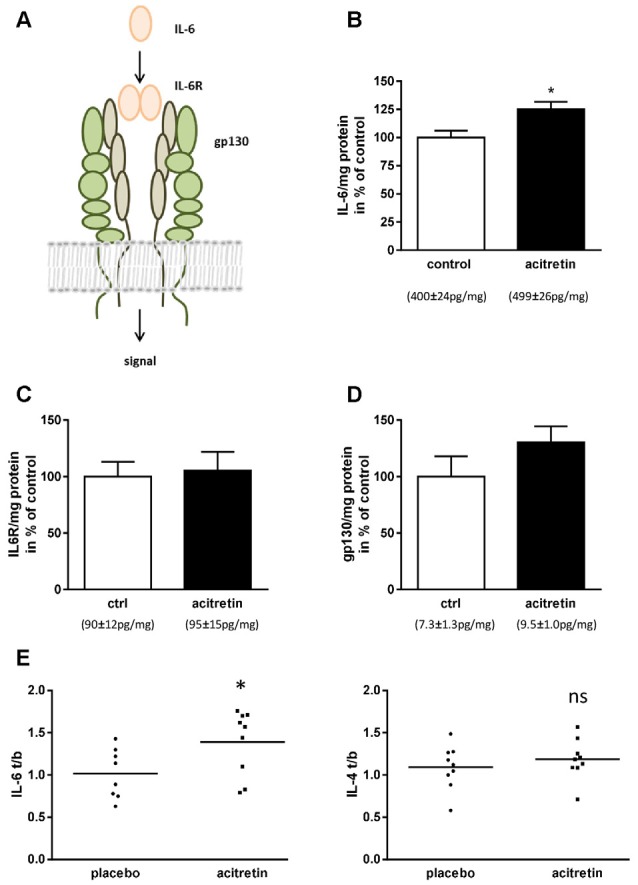
Impact of acitretin-treatment on Interleukin-6 (IL-6) signaling in murine cortex and human CSF. IL-6 dimers bind to IL-6 receptor and, together with gp130, induce downstream classical signaling **(A)**. 5xFAD mice were treated as depicted in Figure 1 and brain homogenates analyzed for IL-6 (**B**; means indicated), IL-6 receptor (IL-6R, **C**), and gp130 (**D**). *n* = 5 per group; Student’s unpaired *t*-test; *p ≤ 0.05). IL-6 and -4 were measured before and after 30 days of acitretin-administration in CSF of Alzheimer patients (**E**, for details of the clinical study see Endres et al., 2014). To account for the inter-individual differences in the small sample size [*n* = 8 (IL-6) and 9 (IL-4) for placebo and *n* = 9 for acitretin], ratios were built [value after treatment/value at baseline (t/b); Student’s unpaired *t*-test; ns, *p* ≥ 0.05; *p ≤ 0.05].

We, therefore, assumed that IL-6 should also be unaltered or diminished in the brains of acitretin-treated mice. Astonishingly, we observed the contrary: IL-6 was increased to 125% of control animals (Figure 2B). IL-6 exerts its biological function *via* two molecules: IL-6 receptor (IL-6R) and gp130 (reviewed e.g., in Garbers and Rose-John, 2018, Figure 2A). Therefore, subsequently to IL-6 quantitation, we analyzed the amount of both receptors. Neither IL-6R nor gp130 was significantly changed on protein level (Figures 2C,D).

### Acitretin-Treatment of Alzheimer Patients Increases CSF Levels of IL-6

In a formerly published clinical study, we demonstrated general feasibility of ADAM10-enhancement by acitretin in patients with mild to moderate AD by measuring APPs-alpha in CSF (Endres et al., 2014). As representatives of anti- and pro-inflammatory markers, IL-4 and IL-6 were measured in CSF (Figure 2E). While IL-4 remained unaffected (placebo group: 1.09 ± 0.09; acitretin group: 1.19 ± 0.08; *p* = 0.44), IL-6 levels increased in the acitretin-group about 40% as compared to placebo-group (placebo group: 1.02 ± 0.10; acitretin group: 1.39 ± 0.13; *p* = 0.04).

## Discussion

Acitretin was able to increase non-amyloidogenic APP processing and AD-related impaired brain function in mice due to short-term treatment. This is in accordance with previous publications reporting on beneficial cognitive effects of retinoids in AD models (Takamura et al., 2017). In addition to its impact on secretase expression, retinoic acid has been shown to control inflammation e.g., in the central nervous system (Raverdeau et al., 2016). Almost all investigated peripheral immune molecules remained unaffected in the acitretin-treated animals despite IL-10. Interestingly, IL-10 has been found to be increased in brain samples derived from humans with intermediate probability of AD in the absence of dementia, designated as resilient (Barroeta-Espar et al., 2019). IL-10 measurements were not considered in the initial clinical study; therefore, the potential effect of acitretin on this IL in human patients still has to be analyzed. IL-6 has been suggested as a plasma biomarker for AD (e.g., Wu et al., 2015) and has also been reported to be repressed by retinoids (Kirchmeyer et al., 2008). However, we observed no change in peripheral IL-6 in mice but only increase in brain IL-6 in both, mice and human patients. In regard to AD, the impact of IL-6 has been found to be multi-faceted: the neurotoxic peptide Aβ induces inflammatory molecules such as IL-6 in glia cultures and in stereotactically injected animals (Forloni et al., 1997; Song et al., 2001). Additionally, the administration of IL-6 to cultured cortical neurons exacerbates toxicity of the peptide (Qiu and Gruol, 2003). However, *in vivo*, IL-6 showed beneficial effects in early stages of disease due to induction of plaque clearance (Chakrabarty et al., 2010) and IL-6-deficient mice showed enhanced neuronal vulnerability in a MPTP-induced Parkinson model (Bolin et al., 2002). The difficulties in deciphering the exact role of IL-6 might be due to the fact that IL-6 may stimulate a response in its target cell in two different manners—the classical signaling and the pathogenicity-driving trans-signaling pathway (Campbell et al., 2014). The first involves binding of IL-6 to the membrane-bound IL-6R, which initiates dimerization of gp130 and subsequent downstream signaling (reviewed in Rothaug et al., 2016). Alternatively, the IL-6 receptor may be shed by proteases such as ADAM10 (Garbers et al., 2011), form a soluble complex with IL-6, and stimulate cells that only express gp130 but not surface-bound IL-6R. Here, we observed an increase in brain IL-6 in mice (and humans) treated with the synthetic retinoid acitretin while IL-6R and gp130 were not significantly impacted. Reports on basal IL-6 secretion or production in brain of 5xFAD mice are somehow controversial (e.g., Ardestani et al., 2017; Mariani et al., 2017); however, another study using also MSD multiplex technique described levels of IL-6 in brain homogenates from these mice comparable to wild type animals at 7 months of age (Chen et al., 2016)—a similar age as for the animals used in our study. This indicates that the comparably small increase of IL-6 due to acitretin-administration may have functional relevance. As the measurement of IL-6R and gp130 was not able to distinguish between soluble and non-soluble forms, we cannot decide which pathway was triggered by acitretin-treatment. Nevertheless, as cognitive function of the mice increased, we could conclude that induction of IL-6 signaling due to acitretin-treatment was not detrimental for the model mice and, therefore, further supports its future therapeutic application in human patients.

## Ethics Statement

This study was carried out in accordance with the recommendations of the Landesärztekammer Rheinland-Pfalz with written informed consent from all subjects. All subjects gave written informed consent in accordance with the Declaration of Helsinki. The protocol was approved by the ethics committee of the Landesärztekammer Rheinland-Pfalz. The trial was monitored by the Interdisciplinary Centre for Clinical Trials Mainz (IZKS, University Medical Centre, Mainz) and registered with ClinicalTrials.gov (NCT01078168). This study was, in regard to animal experiments, carried out in accordance with the recommendations of the European Communities Council Directive regarding care and use of animals for experimental procedures and was approved by local authorities (Landesuntersuchungsamt Rheinland-Pfalz; approval number G14-1-087).

## Author Contributions

MSG quantified CTFs and Aβ, helped with writing the manuscript, interpreted, and visualized the data. NS performed ELISAs, calculated and analyzed the results and conducted the nesting tests. SR-J and CG edited the manuscript and contributed to interpreting the results. CG performed IL-6R and gp130 measurements. AF conducted the clinical trial and edited the manuscript. KE initiated the study, interpreted results, and wrote the manuscript.

## Conflict of Interest Statement

The authors declare that the research was conducted in the absence of any commercial or financial relationships that could be construed as a potential conflict of interest.
